# The Role and Potential Application of IL-12 in the Immune Regulation of Tuberculosis

**DOI:** 10.3390/ijms26073106

**Published:** 2025-03-28

**Authors:** Hangxing Wang, Guiren Ruan, Yuanchun Li, Xiaoqing Liu

**Affiliations:** 1 Division of Infectious Diseases, Department of Internal Medicine, State Key Laboratory of Complex Severe and Rare Disease, Peking Union Medical College Hospital, Chinese Academy of Medical Sciences and Peking Union Medical College, Beijing 100730, China; wanghangxing0111@126.com (H.W.); ruangr@pumch.cn (G.R.); chunyll@163.com (Y.L.); 2 Clinical Epidemiology Unit, Peking Union Medical College, International Clinical Epidemiology Network, Beijing 100730, China; 3 Center for Tuberculosis Research, Chinese Academy of Medical Sciences and Peking Union Medical College, Beijing 100730, China

**Keywords:** tuberculosis, interleukin-12, immunotherapy, molecular mechanisms

## Abstract

Tuberculosis (TB), caused by *Mycobacterium tuberculosis* (*Mtb*), remains a significant global health challenge, affecting millions annually and leading to substantial mortality, particularly in developing countries. The pathogen’s ability to persist latently and evade host immunity, combined with the emergence of drug-resistant strains, underscores the need for innovative therapeutic strategies. This review highlights the crucial role of interleukin-12 (IL-12) in coordinating immune responses against TB, focusing on its potential as an immunotherapy target. IL-12, a key Th1 cytokine, enhances cellular immunity by promoting Th1 cell differentiation and IFN-γ production, vital for *Mtb* clearance. By stimulating cytotoxic T lymphocytes and establishing immune memory, IL-12 supports robust host defense mechanisms. However, the complexity of IL-12 biology, including its roles in pro-inflammatory and regulatory pathways, necessitates a nuanced understanding for effective therapeutic use. Recent studies have shown how IL-12 impacts T cell synapse formation, exosome-mediated bystander activation, and interactions with other cytokines in shaping T cell memory. Genetic defects in the IL-12/IFN-γ axis link to susceptibility to mycobacterial diseases, highlighting its importance in TB immunity. The review also addresses challenges like cytokine imbalances seen in TNF-α/IFN-γ synergy, which exacerbate inflammation, and the implications for IL-12-based interventions. Research into modulating IL-12, including its use as an adjuvant and in recombinant vaccines, promises improved TB treatment outcomes and vaccine efficacy. The review concludes by stressing the need for continued investigation into IL-12’s molecular mechanisms towards precision immunotherapies to combat TB and its complications.

## 1. Introduction

Tuberculosis (TB), a severe chronic infectious disease caused by *Mycobacterium tuberculosis* (*Mtb*), remains a major global health threat [1]. According to the World Health Organization (WHO), TB affects approximately one-quarter of the global population, with tens of millions of new cases diagnosed annually, resulting in nearly one million deaths, predominantly in developing countries [2,3]. Despite advances in TB research and control, significant challenges persist, including the continued spread of the disease and the complexities of treatment. The dormancy of *Mtb* is a formidable challenge; even after standard treatment, dormant bacteria can persist in vivo and relapse unpredictably. This underscores the inadequacy of current treatment regimens in eradicating all bacteria, especially those in a dormant state, thus highlighting the need for new therapies targeting these latent bacteria [4]. Furthermore, the emergence of multidrug-resistant (MDR) and extensively drug-resistant (XDR) TB compounds the difficulty of treatment [5,6]. Consequently, there is an urgent need for innovative treatment strategies, including immunotherapy, to enhance the host’s ability to eliminate bacteria, reduce recurrence risk, and improve overall treatment outcomes.

**Figure 1:**
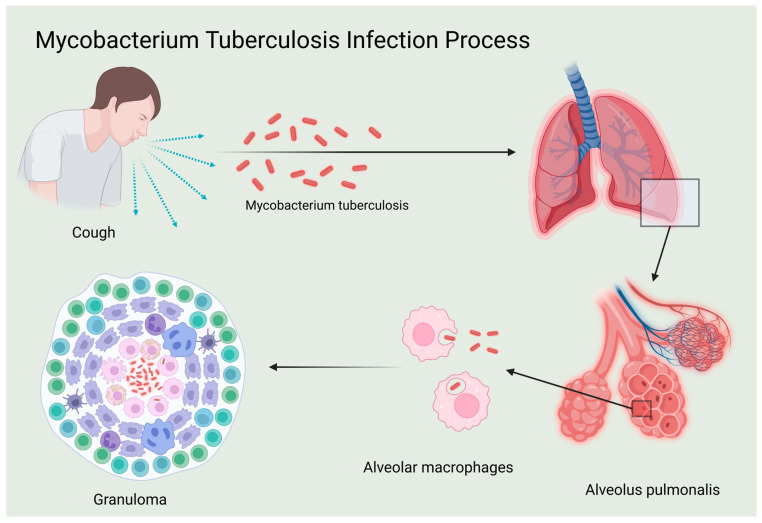
Mycobacterium tuberculosis infection process.

At the immunological level, the host’s immune system plays a crucial role in combating *Mtb*. TB initially infects macrophages and dendritic cells, impairing their antigen-presenting capabilities and leading to latency and potential recurrence. Most infected individuals develop latent tuberculosis infection (LTBI) [7]. During infection, the pathogen can evade the immune response and become dormant within lesions, maintaining a host–pathogen balance through granuloma formation. Resurgence of *Mtb* may occur when host immunity is compromised, with approximately 5% to 15% of LTBI cases progressing to active tuberculosis (ATB) [8]. Following infection, macrophages and dendritic cells release cytokines such as TNF-α, IL-1, IL-6, and IL-12, which are crucial in regulating immune response [9]. IL-12 is a critical cytokine that activates and enhances innate and cellular immune responses, notably by promoting the Th1 cellular immune response through stimulating Th1 cells to release interferon-gamma (IFN-γ), thereby enhancing the host’s ability to clear *Mtb*. In addition to promoting Th1 cell activity, IL-12 indirectly activates cytotoxic T lymphocytes (CTLs), which directly kill infected cells. Cellular immunity also involves the formation of immune memory, enabling a quicker and more effective response to subsequent infections by the same pathogen [10]. Therefore, IL-12 is pivotal in the immune response against Mtb and is crucial for controlling and eliminating TB bacteria (Figure 1).

In recent years, researchers have made significant efforts to deepen the understanding of the immunological mechanisms underlying tuberculosis, particularly the role of IL-12 in immune regulation. Further investigation into the function and regulation of IL-12 holds promise for developing more effective TB treatment and prevention strategies [11,12]. This paper aims to systematically explore the critical role and potential applications of IL-12 in the immune regulation of tuberculosis, providing new avenues for treatment and prevention.

## 2. Overview of IL-12

### 2.1. Structure and Molecular Biological Characteristics of IL-12 Family

The IL-12 family consists of several cytokines, including IL-12, IL-23, IL-27, and IL-35, which play crucial roles in immune regulation and inflammation. Among them, IL-12 is the most extensively studied for its role in *Mtb* infection.

IL-12 is a heterodimer composed of the p35 and p40 subunits [11], primarily synthesized by antigen-presenting cells (APCs) such as macrophages and dendritic cells. Upon binding to its receptor, IL-12 activates signaling pathways such as JAK-STAT, enhancing Th1 cell differentiation and promoting CTL activity, both of which are vital for the immune response to TB [12,13]. IL-23 shares the p40 subunit with IL-12 but includes a distinct p19 subunit. IL-23 is primarily involved in the differentiation and maintenance of Th17 cells, which are important in inflammatory responses. Although its role in TB is less studied, IL-23 may influence the inflammatory environment during active TB, particularly in cases of chronic inflammation or autoimmune involvement [14,15]. IL-27, composed of p28 and EBI3 subunits, regulates both Th1 and Th17 responses. It is involved in immune homeostasis and can modulate immune responses to prevent excessive inflammation. In the context of TB, IL-27 may play a dual role by promoting Th1 responses while controlling overactive immune reactions that could lead to tissue damage [10,16]. IL-35, a heterodimer formed by p35 and EBI3 subunits [17], is primarily produced by regulatory T cells (Tregs). It exhibits potent immunosuppressive activity and plays a key role in maintaining immune tolerance. IL-35 may contribute to the regulation of immune responses, balancing the need for strong defense against *Mtb* while preventing excessive inflammation that could harm the host [18,19,20,21].

Despite structural and functional disparities among IL-12 family members, their collective involvement significantly influences the body’s immune equilibrium and the onset and progression of various diseases.

### 2.2. IL-12 Production and Signal Transduction

#### 2.2.1. Generation of IL-12

**Figure 2:**
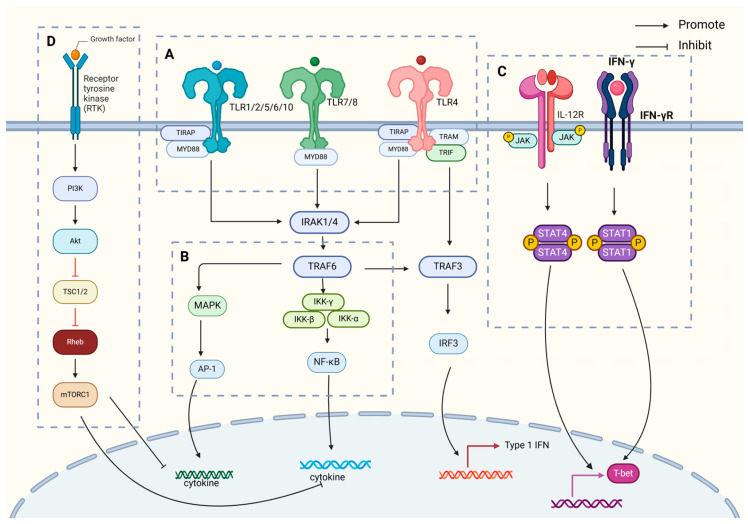
IL-12 generation associated signal transduction pathway diagram. The key signal regulation mechanisms of IL-12 generation mainly include the following. (**A**) TLR-mediated dual-track signal initiation: After TLR recognizes PAMPs in the MyD88-dependent pathway, IRAK family members are summoned to form an activation complex, which activates downstream NF-κB and MAPK signals through TRAF6, directly promoting the transcription and secretion of IL-12 p40/p35 subunits. The TRIF pathway promotes IRF3 phosphorylation by activating TBK1/IKKε, which not only promotes the massive production of IFN-β, but also indirectly enhances the expression of IL-12 p35 subunit. (**B**) CLR-assisted signal enhancement: Recognition of sugar structures on the surface of specific pathogens by CLR activates the SYK/CARD9 axis, which further promotes IL-12 production through NF-κB, MAPK, and IRF pathways. (**C**) Establishment of JAK-STAT positive feedback loop: After IL-12 binds to its receptor, JAK kinase is activated, STAT1/4 is phosphorylated, and ISRE is bound to promote IL-12 expression. (**D**) Fine regulation of PI3K/Akt pathway: The dual regulatory role of PI3K/Akt signal IL-12 expression includes, on the one hand, the inhibition of IL-12 synthesis through mTOR, and on the other hand, promotion of IL-10 production to balance excessive inflammatory response. At the same time, PI3K regulates the expression of various inflammatory factors, including IL-12, by fine-tuning NF-κB activity.

IL-12 is primarily synthesized by APCs, including dendritic cells, macrophages, neutrophils, and B lymphocytes, in response to antigenic stimuli. Its production is regulated by a complex interplay of immune signals, including cytokines such as TNF-α [22] and IL-1β [23], as well as by Th1 cells releasing IFN-γ. This results in a positive feedback loop, amplifying IL-12 production and promoting a robust immune response. IL-12 plays a crucial role in initiating adaptive immunity by supporting Th1 cell differentiation and enhancing CTL activity, both essential for effective immune defense (Figure 2).

#### 2.2.2. Signal Transduction Regulation—Related Signaling Pathways of IL-12 Generation

Major Signaling Pathway 1: Toll-like Receptor (TLR) Signaling Pathway

IL-12 production is regulated by APCs, specifically dendritic cells, through the TLR signaling pathway upon pathogen recognition. TLRs are receptors that bind pathogen-associated molecular patterns (PAMPs) such as bacterial lipopolysaccharides and viral nucleic acids. This engagement activates two key signaling cascades [24,25]: the MyD88-dependent and TRIF-dependent pathways.

In the MyD88-dependent pathway [26], MyD88 recruits IRAK proteins, leading to the activation of TRAF6 and subsequent NF-κB and MAPK signaling pathways. These pathways upregulate the transcription of IL-12 subunits (p40 and p35), promoting IL-12 production, which is crucial for initiating adaptive immunity by promoting Th1 cell differentiation and enhancing NK and CTL activity [27].

In the TRIF-dependent pathway, TRIF activates interferon regulatory factors (IRF3, IRF7) [28,29], enhancing IL-12 production by triggering the activation of TBK1 and IKK-ε, which further induce type I interferons (e.g., IFN-β) and inflammatory factors [30].

Major Signaling Pathway 2: C-Type Lectin Receptor (CLR) Signaling Pathway

The CLR signaling pathway is critical for IL-12 production in response to pathogen recognition by antigen-presenting cells [31]. CLRs, expressed on dendritic cells and macrophages, bind glycosylated pathogen antigens such as bacterial polysaccharides and parasitic glycoproteins [32]. Ligand binding triggers downstream signaling through SYK and CARD9 [33,34], activating NF-κB and MAPK pathways, which enhance IL-12 gene transcription [34,35,36,37].

The activation of CLR also engages transcription factors from NF-κB, AP-1, and IRF families [35,36], which are essential for promoting IL-12 gene expression [37,38].

Major Signaling Pathway 3: Intracellular Signal Transduction Pathway—JAK-STAT

The JAK-STAT pathway is essential for IL-12 signaling. Upon binding to its receptor IL-12R, IL-12 activates JAK kinases [39,40], which are phosphorylate STAT proteins (notably STAT1 and STAT4) [41,42,43]. These phosphorylated STATs form dimers and translocate to the nucleus, binding to IL-12 response elements (ISRE), promoting the transcription of IL-12 genes [42,43,44,45,46,47,48,49]. This pathway not only induces Th1 cell differentiation but also amplifies IFN-γ production, which further enhances IL-12 synthesis, creating a positive feedback loop that strengthens the immune response to pathogens.

Others: PI3K/Akt Signaling Pathway

The PI3K/Akt pathway plays a dual role in regulating IL-12 production. The activation of PI3K by TLRs (especially TLR4) leads to Akt phosphorylation, which modulates inflammatory cytokine production [47,48,49]. On one hand, PI3K/Akt suppresses IL-12 production by targeting mTOR and other effectors [50,51,52], while on the other hand, it promotes the anti-inflammatory cytokine IL-10 [53], which helps regulate excessive inflammation [52]. PI3K also influences NF-κB activity, further regulating IL-12 production [54]. The complex regulation of IL-12 by PI3K highlights its role in balancing pro-inflammatory and anti-inflammatory responses [55].

### 2.3. Modification/Regulation of IL-12 Transcription and Translation

IL-12 production is regulated at multiple levels, including transcriptional and translational processes. Transcription of the IL-12 gene is initiated by signaling through TLRs and CLRs, which activate intracellular pathways like NF-κB and JAK-STAT. These signaling events ensure a prompt response to pathogen-associated signals, with transcription factors such as NF-κB, AP-1, and STATs directly binding to the IL-12 promoter to enhance transcription [56,57].

Co-transcription factors like p300/CBP (transcription coactivating protein 300/CREB binding protein) [54,58] and CBP (CREB binding protein) [59] further modulate IL-12 gene expression through epigenetic modifications, such as acetylation, which enhances gene promoter accessibility and transcriptional efficiency.

At the translational level, IL-12 production is controlled by factors influencing mRNA stability, translation initiation, and post-transcriptional modifications. mRNA stability is regulated by RNA-binding proteins such as AU-rich element-binding proteins [56], which affect IL-12 mRNA degradation [60]. Translation initiation is mediated by factors like eIF-2α, eIF-2B, and eIF-4E [57,61], which ensure efficient mRNA translation [57,60,62]. These processes are regulated by signaling pathways like mTOR and MAPK [63,64], linking extracellular stimuli to translation efficiency.

The mTOR pathway, in particular, is central to controlling ribosome function and adjusting IL-12 production in response to cellular needs, further ensuring efficient immune responses to diverse stimuli.

### 2.4. Secretion of IL-12

The secretion of IL-12 is a carefully orchestrated, multi-step process, beginning with the synthesis of the p35 and p40 subunits, which are transcribed and translated to form IL-12 dimers in the endoplasmic reticulum (ER) [65,66,67]. These dimers are then transported to the Golgi apparatus and early endosomes, where they undergo further modifications [68]. Following this, IL-12 dimers are carried in vesicles to the plasma membrane, where they fuse and release IL-12 into the extracellular space. This process is tightly regulated by various cytokines, including TNF-α and IFN-γ, as well as intercellular interactions, particularly between T cells and dendritic cells. Such regulatory mechanisms ensure the precise modulation of IL-12 secretion, which plays a crucial role in the coordination of immune responses.

### 2.5. Regulation of IL-12

To maintain immune system equilibrium and prevent hyperinflammatory responses, interleukin-12 (IL-12) synthesis and activity are modulated by a spectrum of positive and negative regulators.

(1) Negative Regulation

Direct inhibitors of IL-12 production include IL-10, the transforming growth factor-β (TGF-β), interleukin-4 (IL-4), interleukin-13 (IL-13), and prostaglandin E2 (PGE2) [69]. IL-10 obstructs IL-12 production in antigen-presenting cells by inhibiting NF-κB and STAT4 signaling pathways [70,71]. TGF-β employs the Smad signaling mechanism to hinder IL-12 synthesis, fostering immune tolerance [72]. IL-4 and IL-13 promote Th2 immune responses, diminishing IL-12 production via Stat6 pathway activation [73]. PGE2 limits NF-κB activation through EP2/EP4 receptors, curbing IL-12 production.

Indirect inhibitory mechanisms on IL-12 function are multifaceted and include signaling molecules like suppressor of cytokine signaling 1 (SOCS1) and suppressor of cytokine signaling 3 (SOCS3). These harmful feedback proteins bind to JAK/STAT signaling pathway nodes, diminishing IL-12 signaling efficiency. Immune checkpoint molecules like cytotoxic T-lymphocyte-associated protein 4 (CTLA-4) [74] and programmed cell death protein 1 (PD-1) [75] reduce IL-12 necessity by competitively binding costimulatory molecules, thus depleting T cell function. Although interferon-β (IFN-β) and interferon-α (IFN-α) occasionally boost IL-12 production, they also elevate SOCS protein expression [76], which dampens IL-12 function. TNF-α’s effect varies depending on the physiological and pathological context.

Moreover, upon activation, specific G-protein-coupled receptors, like adenosine A2A receptors, modulate immune cell function, including diminishing dendritic cells’ ability to produce IL-12 [77,78]. These harmful regulatory mechanisms collectively ensure appropriate IL-12 expression, averting excessive inflammation and autoimmune diseases (Table 1).

Exogenous Drug Interventions

Exogenous drug interventions targeting IL-12 and IL-23, such as Ustekinumab [79,80] and Briakinumab [81], effectively block the shared p40 subunit of both cytokines. Consequently, the activation of Th1 and Th17 cells and the production of related pro-inflammatory cytokines are diminished, significantly impacting inflammatory diseases like psoriasis and psoriatic arthritis. Additionally, IL-23-specific inhibitors like Tildrakizumab and Risankizumab [79] indirectly affect IL-12 activity by binding the p19 subunit of IL-23, effectively controlling Th17 cell activation and inflammatory mediator secretion, thereby ameliorating related diseases. Furthermore, Pitrakinra, a competitive antagonist against the IL-4/IL-13 receptor, mainly treats asthma and allergic diseases by competitively binding the IL-12 receptor, mitigating the effects of IL-4 and IL-13, and easing airway inflammation.

(2) Positive regulation

The positive regulation of IL-12 stands as a pivotal process in orchestrating pro-inflammatory responses, particularly those of the Th1 immune pathway, crucial for effective immune surveillance and host defense mechanisms. The regulatory cascade encompasses multifaceted interactions among an array of molecular constituents, cellular entities, and signaling pathways. Initially, the recognition of PAMPs by TLRs, notably TLR-2 [82], TLR-4 [83,84], TLR-5 [85,86], and TLR-9 [87,88], following the detection of bacterial lipopolysaccharides (LPS), flagellin, and diverse microbial components, precipitates downstream signaling events that robustly induce IL-12 production across monocytes, macrophages, and DCs. This T-cell-independent mechanism underpins the rapid innate immune response against early infections. Furthermore, a positive feedback loop involving IFN-γ potentiates IL-12 production, as its secretion by activated NK cells and T cells stimulates mononuclear/macrophages, perpetuating IL-12 synthesis and reinforcing the Th1 immune axis. Additionally, IL-18-mediated activation of NF-κB and MAPK signaling cascades augments IL-12 transcription and expression, intensifying IL-12’s biosynthesis and functional efficacy, and further bolstering Th1 immune response development. Despite the canonical categorization of IL-4 and IL-13 as Th2 cytokines, their nuanced interplay with dendritic cells can facilitate IL-12 subunit transcription, notably p40 and p35, thereby enhancing IL-12 production, notably in the later phases of treatment [89]. This interleukin-induced modulation, complemented by intercellular interactions such as CD40-CD40 ligand (CD40L) engagement between T cells and dendritic cells or macrophages [90,91], fosters IL-12 synthesis, mainly through the preferential induction of p35 gene transcription in dendritic cells, thereby promoting IL-12 heterodimer assembly. In sum, the positive regulation of IL-12 epitomizes a finely tuned interplay of signaling molecules and cellular interactions, safeguarding adequate pathogen clearance and immune homeostasis (Table 2).

## 3. The Regulatory Mechanism of IL-12 on Immune Cells

IL-12 stands as a pivotal regulator in both adaptive and innate immune responses, exerting a profound influence on the activation and differentiation of Th1 cells. Firstly, IL-12 assumes a guiding role in driving Th1 cell differentiation [92], first demonstrated in IL-12p40-deficient murine models [93] and further characterized through STAT4-dependent signaling pathways [94].

Secondly, IL-12 contributes to the sustained proliferation and heightened activity of Th1 cells. Following differentiation, Th1 cells predominantly secrete IFN-γ, and IL-12 presence enhances IFN-γ production, establishing a reinforcing feedback loop that amplifies Th1 cell expansion and maintains their heightened activity. Additionally, IL-12 is crucial in preserving the Th1/Th2 cell balance by impeding Th2 cell differentiation [95]. Given the proclivity of Th1 cell-mediated responses towards cell-mediated immunity, their role is particularly pertinent in combating intracellular infections such as *Mtb* and hepatitis B virus (HBV) [96]. Noteworthy associations have been elucidated between IL-12 levels and serum HBV DNA content in patients with chronic hepatitis B, underscoring its clinical significance [97]. Moreover, IL-12 exerts regulatory influence over Th1/Th2 class cytokine production by peripheral blood mononuclear cells (PBMC), further emphasizing its integral role in immune homeostasis.

IL-12 plays a multifaceted role in regulating the function of NK cells, underscoring its significance in immune modulation [98]. Firstly, IL-12 directly or indirectly enhances NK cell activity, fostering proliferation and augmenting cytotoxicity, mainly when NK cells interact with APCs expressing IL-12. This interaction potentiates NK cell-mediated destruction of virus-infected and tumor cells, bolstering immune surveillance mechanisms. Secondly, IL-12 stimulation induces NK cells and selected lymphocytes, notably CD4+ Th1 cells, to secrete IFN-γ. Moreover, IL-12 signals, in concert with additional costimulatory cues such as IL-15 or TLR agonists [99], synergistically enhance NK cell-killing activity, amplifying their effector functions against pathogens and malignancies. Finally, the IL-12-mediated induction of IFN-γ production is a crucial determinant in steering the Th1 type immune response [100], vital for combating intracellular parasites and specific tumor types.

Under the regulatory influence of IL-12, macrophages demonstrate heightened activation properties, substantially enhancing their phagocytic and bactericidal capabilities [101,102]. Macrophages, adept at pathogen engulfment and destruction, further potentiate their cytotoxicity against infected cells by upregulating the production and release of pro-inflammatory mediators such as TNF-α and nitric oxide (NO) [103]. Concurrently, IL-12 optimizes macrophage antigen presentation proficiency, enabling these activated cells to intricately process and relay phagocytosed pathogen antigenic information to immune effectors like T cells, thereby augmenting overall immune responses. While facilitating the prompt initiation of inflammatory cascades and bolstering macrophage-mediated pathogen clearance, IL-12 also exerts precise control over the magnitude and duration of inflammatory reactions. This timely modulation ensures the inflammatory response remains regulated, preventing excessive tissue damage and maintaining immune homeostasis [104].

IL-12 plays a crucial role in the activation, differentiation, proliferation, and functional enhancement of cytotoxic T lymphocytes (CTLs, also known as CD8+ T cells), as well as in memory formation and tissue localization, underscoring its central importance in antiviral and antitumor immunity [105,106]. IL-12 synergizes with T cell receptor (TCR) signaling and costimulatory molecules to facilitate the differentiation of naïve CD8+ T cells into effector CTLs. This cytokine enhances the cytotoxic capacity of CTLs by upregulating the expression of lytic molecules such as perforin and granzyme B, thereby improving their efficacy in targeting virus-infected and tumor cells. Furthermore, IL-12 significantly induces CTLs to produce large amounts of IFN-γ [107], a potent antiviral and antitumor cytokine that not only directly inhibits pathogen replication and tumor growth but also exerts a wide range of immunoregulatory effects. These effects include enhancing macrophage antigen presentation, upregulating major histocompatibility complex (MHC) molecules, and promoting NK cell activation, thereby amplifying the overall immune response against pathogens and tumors. IL-12 is also instrumental in shaping the memory phenotype of CTLs, facilitating the formation of long-lasting immune memory [108]. CTLs stimulated by IL-12 can differentiate into memory T cells, which persist in the host and rapidly mount robust immune responses upon re-exposure to the same or similar antigens, providing durable protection. Additionally, IL-12 influences the migration and infiltration of CTLs into inflammatory sites or tumor microenvironments by modulating the expression of adhesion molecules and chemokine receptors, ensuring targeted cytotoxic effects where needed. Despite potential systemic toxicity issues in therapeutic applications, the pivotal role of IL-12 in immune regulation underscores its importance. Ongoing research aims to harness IL-12 or its analogs to enhance CTL activity safely and effectively, highlighting its potential in immunotherapeutic strategies.

## 4. Role of IL-12 in the Immune Response to Tuberculosis

IL-12, as a key pro-inflammatory cytokine, occupies a central position in the immune response to tuberculosis, and its normal function directly affects host resistance to *Mtb* infection and disease progression.

### 4.1. Influence of IL-12 in TB Pathogenesis

A. Susceptibility and disease progression: Genetic studies have revealed significant associations between polymorphisms in IL-12 and their receptor genes and individual TB susceptibility [109]. Specifically, defective or dysfunctional IL-12 genes may lead to reduced IL-12 biosynthesis, which in turn weakens the host’s innate immune defense against *Mtb* and increases the risk of infection [110]. After the onset of infection, changes in IL-12 levels reflect the strength and direction of the immune response. Low levels of IL-12 are often associated with elevated disease activity, deterioration, and poor response to therapy. Conversely, high levels of IL-12 tend to portend a better clinical prognosis, possibly stemming from a more effective anti-*Mtb* immune response and inflammatory control [111].

B. Immunomodulation and pathological processes: The central role of IL-12 in TB immunomodulation is reflected in its dual activation of the natural and adaptive immune systems. First, IL-12 stimulates the activation of macrophages and dendritic cells, enhancing their ability to phagocytose and kill *Mtb*. This is mainly through the upregulation of the NADPH oxidase-mediated oxidative burst [112], which induces the expression of acidified lysozyme and nitric oxide synthase (iNOS) [113], thereby promoting NO production, all of which are important mechanisms for the direct inhibition of *Mtb* growth. Second, IL-12 critically promotes Th1 cell differentiation and enhances macrophage function by stimulating IFN-γ production while initiating the synergistic secretion of Th1-type cytokines. In addition, IL-12 maintains an inflammatory microenvironment that favors Th1-type immune responses and prevents lung tissue damage caused by excessive inflammation, such as cavity formation and fibrosis, by suppressing Th2-type responses and the activity of regulatory T cells (Tregs) [114]. IL-12 deficiency may lead to an imbalance of immune responses, manifested by insufficient anti-*Mtb* immune effects or inflammatory responses that are excessive, exacerbating pathological changes in the lungs [115].

C. Therapeutic intervention and immune enhancement: IL-12 is widely regarded as a promising immunotherapeutic target given its key role in the immune response to TB. Currently, therapeutic strategies targeting IL-12 are divided into two main directions: First, direct supplementation of IL-12 to enhance immune response, including the use of recombinant IL-12 protein therapy or gene therapy to increase the level of IL-12 in the body, enhance the immune response of Th1 cells, and promote *Mtb* clearance. Preclinical studies, including seminal work by JoAnn Flynn’s group in murine TB models [116], have demonstrated that recombinant IL-12 administration significantly reduces pulmonary bacterial burden (1.5 log10 CFU reduction) and enhances granuloma resolution through IFN-γ-dependent macrophage activation, and IL-12 is even expected to be used for the prevention of TB relapse [117,118,119]. On the other hand, the modulation of IL-12-related signaling pathways by small molecule drugs or biologics is also a hot research topic. For example, the development of agonists targeting the IL-12 receptor or its downstream signaling molecules such as STAT4 can specifically enhance IL-12 signaling and promote immune cell function without increasing the risk of systemic inflammatory responses [120]. In addition, studies targeting negative regulators of IL-12 production, such as inhibitors of IL-10 or SOCS proteins [76,121], may help to deregulate the inhibition of IL-12 production and thus indirectly enhance its immune effects.

### 4.2. Interaction Mechanism Between IL-12 and Mtb

**Figure 3:**
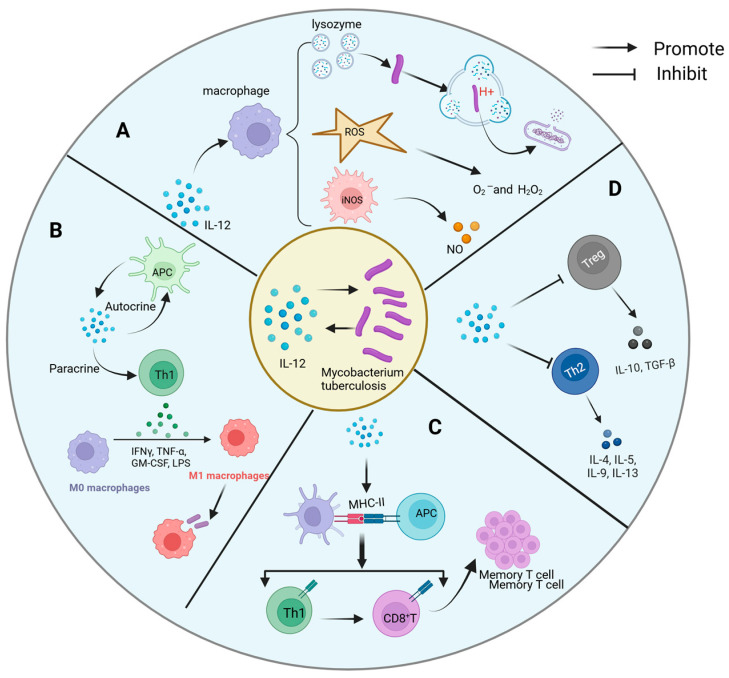
Mechanism of interaction between IL-12 and Mycobacterium tuberculosis. The complex interaction mechanism between IL-12 and Mtb includes the following: (**A**) direct antibacterial action: IL-12 indirectly enhances the defense against Mtb by promoting macrophage activation. It promotes the production of ROS and NO, as well as the activation of acidifying lysozyme, mechanisms that work together to disrupt the structure of Mtb, inhibit its growth, and optimize the host’s first line of defense against Mtb. (**B**) Activation and differentiation of immune cells: IL-12 activates Th1 through autocrine and paracrine, resulting in the production of a large amount of IFN-γ, which strengthens the phagocytosis and bactericidal ability of macrophages. (**C**) Antigen presentation and memory formation: IL-12 can promote the maturation of APCs and enhance the expression of MHC molecules, activate specific Th1 and CD8+ T cells, and lay the foundation for the establishment of long-term immune memory. (**D**) Fine regulation of inflammatory microenvironment: IL-12 not only simply promotes Th1 response, but also inhibits Th2 response and Treg activity, preventing the imbalance of immune response, so as to achieve effective control of Mtb infection.

The interaction mechanism between IL-12 and *Mtb* is intricate and multifaceted, encompassing both direct and indirect pathways of influence (Figure 3).

A. Direct antibacterial activity

Regarding direct antibacterial activity, although IL-12 lacks inherent bactericidal capabilities, it significantly potentiates the effectiveness of immune cells against *Mtb* by orchestrating macrophage activation. Mechanistically, IL-12 operates on multiple fronts: Firstly, it fosters the generation of reactive oxygen species (ROS) within macrophages, including superoxide anions and hydrogen peroxide. These ROS molecules exert oxidative stress on *Mtb*, leading to structural damage and inhibition of bacterial proliferation. Secondly, IL-12 facilitates the upregulation of acidifying lysozyme expression and activity, thereby facilitating the degradation of Mtb’s cell wall components, including its characteristic mycobacteria acid, within acidic phagolysosomes. This disruption compromises bacterial integrity. Additionally, IL-12 stimulates the synthesis of NO, which disrupts essential life processes of *Mtb*, such as DNA synthesis, energy metabolism, and protein synthesis. Collaborating with ROS and other antibacterial mechanisms, NO amplifies the inhibitory effect on *Mtb* proliferation [122]. Through these concerted cellular activities, IL-12 fortifies macrophage defense mechanisms against *Mtb*, fundamentally restraining bacterial replication and survival within host cells.

B. Immune cell activation and differentiation

IL-12 assumes a pivotal role in orchestrating the adaptive immune response, particularly in the activation and differentiation of T helper 1 (Th1) cells, which are crucial players in combating *Mtb*. Through diverse pathways, IL-12 potentiates the functionality of Th1 cells and mobilizes other immune effectors against *Mtb*. Primarily, IL-12 elicits robust IFN-γ production by Th1 cells, a pivotal cytokine in anti-*Mtb* immunity. IFN-γ exerts multifaceted effects, augmenting the antimicrobial prowess of macrophages by enhancing *Mtb* clearance mechanisms. This includes upregulating major histocompatibility complex class II (MHC-II) molecules, bolstering phagocytic activity, and promoting ROS and NO production within macrophages [123].

Furthermore, IL-12 sustains the proliferation and viability of Th1 cells via autocrine and paracrine pathways, ensuring continuous IFN-γ secretion. Additionally, IL-12 fosters immune memory formation by enhancing the expression of costimulatory molecules on Th1 cell surfaces, facilitating their interaction with APCs. Notably, Th1 cytokines such as IFN-γ and TNF-α, induced by IL-12, not only directly impact macrophage function but also activate cytotoxic CD8+ T cells and NK cells, bolstering the collective immune response against *Mtb*.

C. Antigen presentation and immune memory

IL-12 plays a crucial role in enhancing Th1 cell differentiation and IFN-γ production, both of which are essential for controlling intracellular pathogens [124]. Additionally, recent studies have demonstrated that IL-12 can modulate the activity of regulatory T cells, further influencing the immune response. For instance, a study [125] has shown that IL-12 not only influences the differentiation of Th1 cells but also plays a significant role in regulating immune tolerance and modulating inflammatory responses. IL-12 exerts a profound influence on antigen presentation and immune memory, which are pivotal components of the host defense against *Mtb*. By promoting DC maturation and enhancing the expression of major histocompatibility complex MHC class I and II molecules, IL-12 significantly augments the efficiency of *Mtb* antigen presentation. This orchestrated process ensures the effective activation of specific T cells, particularly Th1 and CD8+ T cells, upon the initial *Mtb* encounter. Subsequently, following the resolution of infection, the Th1-polarized immune milieu fostered by IL-12 facilitates the generation and perpetuation of memory T cells [108]. These enduring memory T cells persist within the host for extended durations, poised to mount rapid and robust immune responses upon re-exposure to *Mtb*. This mechanism effectively curtails or prevents tuberculosis recurrence, underscoring the crucial role of IL-12 in long-term immune memory maintenance and host defense against *Mtb*.

D. Shaping of the inflammatory microenvironment

IL-12 plays a pivotal role in shaping the inflammatory microenvironment by finely regulating cytokine networks, thereby ensuring dominance of the Th1-type immune response while concurrently suppressing Th2-type responses [95] and regulatory T cell (Tregs) activity [114]. This intricate regulatory mechanism, documented in prior studies, mitigates immunosuppression and tissue destruction, thus fostering effective anti-*Mtb* immune response. By orchestrating this targeted modulation of the inflammatory milieu, IL-12 prevents lung damage resulting from excessive inflammation, while concurrently promoting the efficient clearance of *Mtb* within the lesion site. This nuanced regulatory function underscores the critical importance of IL-12 in maintaining immune homeostasis and optimizing host defense mechanisms against *Mtb* infection.

### 4.3. IL-12 and Drug-Resistance Mycobacterium Tuberculosis

The molecular interplay between IL-12 and *Mtb* drug resistance is rooted in the cytokine’s dual role as a regulator of host immunity and a target of pathogen-driven immune evasion. IL-12, produced by DCs and macrophages upon *Mtb* recognition, drives Th1 differentiation via STAT4 signaling, leading to IFN-γ production—a critical mediator of macrophage bactericidal activity through NO synthesis and autophagy activation. However, *Mtb* employs sophisticated mechanisms to suppress IL-12 signaling, indirectly fostering drug resistance. For instance, *Mtb*-derived LAM binds TLR2 to inhibit DCs maturation, reducing IL-12 secretion and skewing the immune response toward an IL-10-dominated anti-inflammatory state. This immunosuppressive shift not only impairs bacterial clearance but also creates a permissive environment for subpopulations of *Mtb* to persist under suboptimal drug concentrations, enabling the accumulation of resistance-conferring mutations [126,127]. Notably, IL-12 deficiency exacerbates metabolic dysregulation in macrophages, such as heightened glycolysis via HIF-1αand impaired mitochondrial oxidative phosphorylation, which *Mtb* exploits to evade antimicrobial stress. Furthermore, *Mtb* strains with drug-resistance mutations exhibit altered cell wall lipids, which directly suppress IL-12 transcription by recruiting HDACs to the IL12B promoter, thereby dampening Th1 responses. Conversely, IL-12 enhances antigen presentation by upregulating MHC-II expression and counteracts IL-10-mediated inhibition of NF-κB, restoring pro-inflammatory signaling necessary for drug efficacy [128]. Emerging evidence also highlights IL-12’s role in modulating non-coding RNAs, which regulate pathways like autophagy and apoptosis, processes critical for eliminating drug-tolerant *Mtb* persisters [129,130]. These molecular interactions underscore IL-12 as a pivotal node in the host–pathogen conflict, where its suppression by *Mtb* not only compromises immune control but also indirectly perpetuates drug resistance by fostering bacterial survival and genetic adaptation [131]. Therapeutic strategies targeting IL-12 restoration—such as nanoparticle-delivered IL-12 mRNA or IL-10 receptor antagonists—may thus synergize with existing antibiotics to disrupt this vicious cycle [132].

## 5. Clinical Application of IL-12 in Diagnosis, Treatment, and Prognosis Evaluation of Tuberculosis

### 5.1. Diagnosis

A. Importance of IL-12 in Tuberculosis Diagnosis

Previous investigations have established a correlation between IL-12 levels and TB severity and treatment outcomes [133]. Consequently, assessing IL-12 concentrations in TB patients’ serum or cerebrospinal fluid holds promise as a valuable diagnostic tool. Such measurements offer insightful data for evaluating disease activity, prognostication, and tailoring individualized treatment strategies.

B. Potential Role of IL-12 and Other Cytokines in Tuberculosis Diagnosis

Ren et al. observed elevated serum levels of various cytokines, including IL-1β, IL-6, IL-8, IL-12p70, TNF-α, and IFN-γ, in patients with tuberculosis and tuberculosis pneumonia compared to healthy individuals. Particularly noteworthy was the significant elevation observed in patients with tuberculosis pneumonia. This suggests that a comprehensive assessment of multiple factors may enhance the accuracy of TB and chronic pulmonary aspergillosis (CPA) diagnosis [134]. Such multifactorial testing approaches not only aid in precise disease diagnosis but also deepen insights into the intricate immune responses elicited during infections with *Mtb* and Aspergillus fumigatus [135].

C. Potential Use of Host Biomarkers in Tuberculosis Diagnosis

In tuberculosis (TB) diagnosis, a recent study investigating the utility of host biomarkers in pulmonary tuberculosis (PTB) diagnosis revealed notable findings [136]. Comparative analysis of serum samples from 55 PTB patients and 106 individuals with other respiratory diseases (ORDs) unveiled significant differences in several biomarkers, including IP10, IL6, IL2, IL1β, TNF-α, IFN-γ, and IL12p70 between the two groups. Notably, a biomarker combination comprising IP10, IL6, TNF-α, IL1β, IL1ra, and IL12p70 exhibited exceptional diagnostic performance in PTB diagnosis. This had an area under the receiver operating characteristic (ROC) curve reaching up to 90%, indicating its remarkable diagnostic utility. This discovery offers a valuable repertoire of biomarkers for the swift screening of active TB, poised to play a pivotal adjunctive diagnostic role in clinical settings.

Moreover, Yu et al. have substantiated through animal experimental studies that *Mtb* infection in mice leads to elevated levels of IL-35 and inducible T regulatory type 35 (iTr35) subsets, concomitant with increased bacterial burden and lung lesions. IL-35 and iTr35 cells may exert immunosuppressive effects in chronic *Mtb* infection [137]. In patients with ATB, leukocytes and peripheral blood mononuclear cells exhibited significantly increased mRNA expression of IL-35 and its subunits p35 and EBI3. Following anti-TB drug treatment, serum IL-35 levels and p35 or EBI3 expression showed a reduction, suggesting the potential of IL-35 as a biomarker for TB immune status and prognosis assessment [19,20].

D. Superiority of IL-27 in the Diagnosis of Tuberculous Pleurisy

As a member of the IL-12 cytokine family, IL-27 exhibits unique diagnostic utility in tuberculous pleurisy. Unlike IL-12’s role in driving Th1 immunity, IL-27 regulates immune balance while serving as a biomarker for effusion differentiation, which exhibits notable sensitivity and specificity in diagnosing tuberculous pleurisy, as evidenced by meta-analytical investigations [138]. Specifically, IL-27 demonstrates remarkable efficacy in distinguishing tuberculous pleural effusion from malignant pleural effusion. Furthermore, when combined with IFN-γ and/or adenosine deaminase (ADA), IL-27 markedly enhances diagnostic sensitivity and specificity to 100%, further substantiating its central role in tuberculous pleurisy diagnosis. Domestic research endeavors corroborate the diagnostic efficacy of IL-27, either as a standalone marker or in conjunction with IFN-γ and ADA, not only in accurately identifying tuberculous pleurisy but also in notably augmenting the specificity of diagnostic assays. These collective findings underscore the heightened diagnostic value of IL-27 compared to alternative markers in the context of tuberculous pleurisy diagnosis [139].

E. Diagnostic Value of IL-12 in Diseases Associated with Immune Dysfunction

The diagnostic significance of IL-12 in immune dysfunction is profound, particularly in patients afflicted with primary immunodeficiency syndrome (PIDs) characterized by mutations in IL-12 and IFN-γ-related immune pathway genes. Such mutations markedly elevate susceptibility to nontuberculous mycobacteriosis (NTM disease). These insights underscore the pivotal role of IL-12 in identifying and understanding immune dysregulation, offering valuable implications for diagnostic strategies and therapeutic interventions in afflicted individuals [140,141,142].

### 5.2. Treatment

The clinical application of IL-12 in tuberculosis treatment has yet to be directly incorporated into conventional therapeutic regimens. However, its potential role in immune modulation and antituberculosis therapy has garnered extensive investigation and exploration.

A. Immuno-enhancer and Adjuvant Combination Therapy: IL-12 exhibits the capacity to bolster Th1 type immune responses, augment the activity of NK cells and T cells, and induce IFN-γ production, thereby enhancing the host’s defense against *Mtb*. Theoretically, supplementing IL-12 or enhancing its activity could bolster the immune-mediated clearance of TB, particularly in immunocompromised or drug-resistant TB patients. A 2021 study [143] explored the potential of IL-12 in improving CD4+ T cell function, which, in turn, may enhance the efficacy of antibiotic therapy in TB. In a study by Nolt and Flynn [144], IL-12 was administered to C57BL/6 mice via respiratory aerosolization over eight weeks. These mice were infected with *Mtb*. Results demonstrated that IL-12 significantly improved the survival rate of CD4+ T-cell deficient mice and reduced bacterial load, suggesting the potential of IL-12 to directly or indirectly impede *Mtb* growth and ameliorate disease status. Moreover, IL-12 was found to upregulate the expression of *Mtb*-specific IL-21 at TB lesion sites, notably enhancing the proportion of IL-21+IFN-γ+CD4+ T cells, indicative of its role in bolstering local immune responses for effective *Mtb* control [145]. While IL-12 currently does not serve as a frontline drug for TB treatment, investigations have explored its potential utility in combination with traditional anti-TB drugs to expedite recovery and diminish bacterial load by fortifying the host’s immune response. Such approaches hold promise for addressing drug-resistant or complex TB cases.

B. Prevention of Recurrence and Transmission: Modulating cytokine levels such as IL-12 contribute to diminishing the risk of tuberculosis recurrence post-cure or inhibiting the potential for latent TB bacteria to reactivate within the body. The utilization of IL-12 holds promise in preventing TB recurrence, thereby mitigating TB dissemination and prevalence within the community.

C. The Potential Role of IL-12 in Tuberculosis Treatment: While the role of IL-12 in conventional CD4+ T cell immunity is well-established, its impact on the non-CD4+-expressing subset of γδT cells T cells, particularly Vγ2Vδ2 T cells, remains incompletely elucidated [146,147]. Vγ2Vδ2 T cells play a pivotal role in combating *Mtb* infection, as they recognize *Mtb* metabolites isopentenyl pyrophosphate (IPP) and hydroxy-methylbutenyl pyrophosphate (HMBPP) and swiftly secrete Th1 cytokines like IFN-γ and TNF-α [147]. Recent investigations have unveiled that IL-12 may foster the expansion and differentiation of HMBPP-activated Vγ2Vδ2 T cells via PI3K/AKT and STAT4 signaling pathways, endowing them with heightened antibacterial activity and memory phenotype and facilitating the release of diverse antibacterial cytokines and cytotoxic particles. Thus, IL-12 exerts a direct and indirect beneficial impact on antituberculosis immunity [148,149].

### 5.3. Prognosis Assessment

In a clinical study conducted in India to assess the changes in serum biomarkers among patients with active tuberculosis following treatment with antituberculosis drugs, it was observed that individuals with pulmonary tuberculosis (APTB) exhibited elevated serum levels of pro-inflammatory cytokines, including IL-12p40, IFN-γ, TNF-α, IL-1β, and IL-6 at baseline compared to healthy controls (*p* < 0.01). After six months of antituberculosis drug therapy, the levels of IFN-γ, TNF-α, IL-1β, IL-12p40, and IL-6 in APTB patients approached values observed in the healthy population. This trend suggests a potential role for IL-12 in the prognostic evaluation of ATB, however, further investigations are warranted to substantiate these findings [150]. Additionally, the post-treatment expression of serum IL-35 was observed to decrease compared to pre-treatment levels, implicating a potential role for IL-35 in the assessment of tuberculosis prognosis [20,151].

### 5.4. Prevention

In striving for an effective TB vaccine strategy, the aim is to prevent both primary infection and post-exposure disease, halt the reactivation of latent infection, and act as a complement to standard TB treatment. Currently, novel TB vaccines undergoing clinical trials encompass a variety of types, including viral vector vaccines, recombinant protein/adjuvant vaccines, whole cell/extract vaccines, attenuated/recombinant live vaccines, and DNA vaccines. Notably, IL-12 has shown promising results when employed as an adjuvant expressed via a plasmid carrier. Its incorporation into a specific DNA promoter as an adjuvant added to the MVA85A vaccine notably augments the immune response to *Mtb* infection [152]. This adjuvant strategy bolsters vaccine protection by enhancing the vaccine-induced Th1-type immune response, which is particularly beneficial for individuals with diminished responsiveness to the BCG vaccine. Furthermore, the synergistic combination of IL-12 with other adjuvants, such as TLR agonists and alum, holds the potential to optimize vaccine formulations, enhance vaccine immunogenicity, and reduce vaccine dose or frequency requirements [153,154].

### 5.5. Disease and Susceptibility to Tuberculosis

In addition to well-established risk factors such as diabetes [155,156] and HIV [157], other immune-related diseases may heighten susceptibility to *Mtb* infection. Autoimmune conditions like rheumatoid arthritis and systemic lupus erythematosus, often managed with immunosuppressive therapies, compromise immune function, thereby increasing tuberculosis susceptibility. Furthermore, chronic lung ailments, notably chronic obstructive pulmonary disease (COPD) [158], represent a significant risk factor for TB susceptibility due to lung tissue damage and impaired airway clearance.

Alterations in IL-12 levels play a pivotal role in host immune function in these diseases. Metabolic dysregulation in diabetic patients notably impairs the function of innate immune cells, resulting in reduced secretion of key cytokines like IL-1β, IL-12, and IL-18 and diminished IFN-γ response upon stimulation. Such immunological changes contribute to heightened tuberculosis vulnerability, likely stemming from chronic inflammation and metabolic irregularities inherent in the disease state. In individuals with AIDS, HIV-induced immune compromise may inhibit IL-12 production, further exacerbating susceptibility to TB.

The immune system often operates in a hyperactive or compromised state for patients with other immune disorders and COPD, potentially impacting IL-12 production. Consequently, variations in IL-12 levels may significantly influence host resistance to *Mtb*, escalating infection risk and disease progression. Notably, for patients with Mendelian susceptibility to mycobacterial disease (MSMD) [159,160], while the genetic etiology remains unclear for some, IL-12 and IL-23-dependent IFN-γ immunity emerge as critical for *Mtb* resistance, offering invaluable avenues for further investigation.

### 5.6. Potential Strategies for Leveraging IL-12 Against Mtb Drug Resistance

Combination of Immunotherapy with Traditional Antibiotic Treatment: Given that IL-12 significantly enhances the host’s immune response against Mtb, it is proposed to use IL-12 as an adjunctive therapy in combination with existing anti-TB drugs. This approach aims to improve treatment efficacy and reduce the emergence of drug-resistant strains. For example, IL-12 expressed via a plasmid carrier has been shown to enhance vaccine-induced Th1-type immune responses [161]. Personalized approaches may involve monitoring IL-12 levels and adjusting treatments accordingly [162]. Development of Novel IL-12 Agonists: Exploration of new small molecule drugs or vaccine components that specifically enhance IL-12 signaling pathways could provide more effective immune protection without causing unnecessary systemic inflammation. A novel vaccine (HVJ-liposome/HSP65 DNA+IL-12 DNA) has been evaluated against TB using the cynomolgus monkey model, showing promise in enhancing immune responses [163].

## 6. Summary

In summary, IL-12 emerges as a pivotal player in the immune modulation of tuberculosis, as illustrated in Figure 1, which shows the process of *Mycobacterium tuberculosis* infection. This infection process is critical to understanding how IL-12 mediates immune responses, including the differentiation of Th1 cells, enhancement of cytotoxic T lymphocyte function, and promotion of immune memory formation. The intricate production, secretion, and regulatory mechanisms of IL-12, depicted in Figure 2, profoundly influence the magnitude of immune responses, infection outcomes, and disease progression. The key signal regulation mechanisms of IL-12 generation, shown in Figure 2, highlight the involvement of various signaling pathways. These include TLR-mediated dual-track signal initiation (A), CLR-assisted signal enhancement (B), the establishment of a JAK-STAT positive feedback loop (C), and the fine regulation of the PI3K/Akt pathway (D). Each of these pathways plays a crucial role in IL-12 production and underscores the complexity of immune regulation in tuberculosis.

IL-12’s direct and indirect actions against tuberculosis pathogens, through the facilitation of Th1 cell differentiation, augmentation of cytotoxic T lymphocyte function, and promotion of immune memory formation, underscore its indispensable role in infection containment and pathogen eradication. Furthermore, IL-12’s homeostatic regulation, including its modulation of positive and negative regulators like IL-10 and TGF-β, alongside the advent of drug intervention strategies like IL-12/IL-23 inhibitors, underscore the multifaceted nature of its involvement in immune homeostasis maintenance and therapeutic interventions.

Clinically, IL-12 level fluctuations serve as a crucial indicator for tuberculosis diagnosis, treatment response monitoring, and prognosis assessment. Combined detection with other cytokines enhances diagnostic accuracy, while IL-27 and other IL-12 family members exhibit notable advantages in diagnosing specific TB presentations such as tuberculous pleurisy. These insights enrich our comprehension of TB immune pathophysiology and offer novel avenues for personalized treatment and precision medicine.

In conclusion, as a pivotal immune regulatory molecule, IL-12’s role in tuberculosis elucidates the disease’s immune regulatory network. It furnishes a scientific foundation for novel therapeutic and preventive strategies such as vaccine formulation and immunotherapy optimization [164,165,166,167,168,169]. Future investigations should delve deeper into IL-12’s mechanisms and regulatory network, analyzing its dynamics across various disease stages and populations to craft more efficacious disease control and eradication strategies and bolster global TB prevention and management efforts.

## Figures and Tables

**Table 1:** IL-12 Negative Regulators and Their Mechanisms of Action.

Classification	Name	Mechanism	Effect
Direct Negative Regulation of IL-12	IL-10	Inhibits NF-κB and STAT4 activity, prevents transcription of IL-12 p35 and p40 genes.	Inhibits IL-12 synthesis and secretion, promotes Th2 immune response, inhibits Th1 immune response.
	TGF-β	Inhibits IL-12 transcription via Smad signaling pathway and other non-Smad pathways.	It suppresses IL-12 biosynthesis, maintains immune tolerance, and suppresses the excessive inflammatory response.
	IL-4 and IL-13	Induce bias toward Th2 polarization via Stat6-mediated signaling pathway, inhibiting IL-12 production.	Inhibition of IL-12 production promotes Th2 immune response and diminishes Th1 immune response.
	PGE2	Inhibits NF-κB activation and reduces IL-12 production via EP2 and EP4 receptors.	Regulates IL-12 synthesis to prevent excessive inflammation.
Indirect Negative Regulation of IL-12	SOCS1 and SOCS3	Block IL-12 signaling by binding to JAK kinases or STAT proteins.	Indirectly reduces IL-12 function and regulates cell signaling pathways.
	CTLA-4	Inhibits T-cell activation and proliferation by competitively binding to the costimulatory molecule CD80/CD86.	Indirectly reduces T-cell activity and affects IL-12 demand and effects.
	PD-1	Binds to PD-L1/PD-L2, leading to T-cell depletion and immunosuppression, affecting IL-12 production by antigen-presenting cells.	Indirectly affects IL-12 production and effects, regulating immune responses.
	IFN-β and IFN-α	Affect IL-12 production through complex positive and negative feedback mechanisms, including upregulation of SOCS protein expression.	Indirectly inhibit IL-12 biosynthesis under certain conditions.
	TNF	Promotes IL-12 production under normal conditions, but in chronic inflammation or hyperinflammatory responses, high levels of TNF-α inhibit IL-12 production.	Regulates IL-12 production and immune responses according to conditions.
	GPCRs	Indirectly inhibit IL-12 production by altering the functional state of immune cells.	Inhibit IL-12 production by altering signaling pathways.
Exogenous Drugs	IL-12 and IL-23 inhibitors: Ustekinumab, Briakinumab	Bind to and block the shared p40 subunit of IL-12 and IL-23.	Down-regulate Th1-mediated inflammation by inhibiting IL-12 signaling, effectively controlling diseases dominated by Th1 immune response.
	IL-23 specific inhibitors: Tildrakizumab and Risankizumab	Bind specifically to the p19 subunit of IL-23.	Indirectly affect IL-12 activity, controlling Th17 cell activation and secretion of inflammatory mediators such as IL-17.
	Competitive antagonist of IL-4/IL-13 receptor: Pitrakinra	Competitively binds to IL-12 receptor.	Blocks Th2 immune response, alleviating airway inflammation and reducing IL-4 and IL-13 effects, primarily used in asthma and allergic diseases.

**Table 2:** IL-12 Positive Regulators and Their Mechanisms of Action.

Classification	Name	Mechanism	Effect
Cytokines	IFN-γ	Activates JAK-STAT signaling pathway, promoting IL-12 transcription and synthesis.	Enhances IL-12 expression in macrophages and dendritic cells, fostering Th1 cell response.
	IL-4/IL-13	Exhibits a bimodal effect: initially inhibits p40 production, later strongly enhances p40 production.	Stimulates IL-12 heterodimer production by upregulating transcription of p40 and p35 genes.
	IL-18	Activates IL-18R signaling pathway, enhancing NF-κB and MAPK pathways, leading to increased IL-12 transcription and expression.	Amplifies IL-12 production and bioactivity, synergistically promoting Th1-type immune response.
Pattern Recognition Receptor Agonists	TLRs (TLR-2, -4, -5, -9) agonists	Initiates transcription factors such as NF-κB, AP-1, augmenting IL-12 transcription and synthesis.	Promotes immune response by recognizing PAMPs (e.g., LPS, flagellin), robustly inducing IL-12 production in monocytes/macrophages and DCs without T cells.
	Self-microbial products (bacteria, intracellular parasites, fungi, dsRNA, CpG DNA)	Binds to TLR, triggering transcription factor signaling, promoting IL-12 production.	Significantly boosts IL-12 expression, enhancing immune response.
Immune Cell-to-Cell Interaction	Molecule CD40 Ligand (CD40L, CD154)	CD40L on T cells binds to CD40 on DCs or macrophages, promoting IL-12 expression.	Facilitates optimal IL-12 production through T cell and APC interaction, with CD40 stimulation preferentially inducing p35 gene transcription.
Transcription Factor Cascade Response	IRF Transcription Factors (IRF1, IRF2, IRF5, IRF7, IRF8)	Regulate IL-12 gene transcription, with deficiencies leading to impaired IL-12 expression, affecting p35 and p40 expression.	Key transcriptional regulators ensure timely IL-12 production.

## Data Availability

The authors declare that all data were generated in-house and that no paper mill was used.
